# Alternative Substrates for the Development of Fermented Beverages Analogous to Kombucha: An Integrative Review

**DOI:** 10.3390/foods13111768

**Published:** 2024-06-05

**Authors:** Vinicius Costa Barros, Vanessa Albres Botelho, Renan Campos Chisté

**Affiliations:** 1 Graduate Program of Food Science and Technology (PPGCTA), Institute of Technology (ITEC), Federal University of Pará (UFPA), Belém 66075-110, PA, Brazil; vi_costa_b@outlook.com; 2 Faculty of Food Engineering, Institute of Technology (ITEC), Federal University of Pará (UFPA), Belém 66075-110, PA, Brazil

**Keywords:** kombucha, fermented beverages, food technology, Amazonian herbs

## Abstract

Kombucha is a fermented beverage that originated in China and is spread worldwide today. The infusion of *Camellia sinensis* leaves is mandatory as the substrate to produce kombucha but alternative plant infusions are expected to increase the opportunities to develop new fermented food products analogous to kombucha, with high technological potential and functional properties. This review gathers information regarding promising alternative substrates to produce kombucha-analogous beverages, focusing on plants available in the Amazonia biome. The data from the literature showed a wide range of alternative substrates in increasing expansion, with 37 new substrates being highlighted, of which ~29% are available in the Amazon region. Regarding the technological production of kombucha-analogous beverages, the following were the most frequent conditions: sucrose was the most used carbon/energy source; the infusions were mostly prepared at 90–100 °C, which allowed increased contents of phenolic compounds in the product; and 14 day-fermentation at 25–28 °C was typical. Furthermore, herbs with promising bioactive compound compositions and high antioxidant and antimicrobial properties are usually preferred. This review also brings up gaps in the literature, such as the lack of consistent information about chemical composition, sensory aspects, biological properties, and market strategies for fermented beverages analogous to kombucha produced with alternative substrates. Therefore, investigations aiming to overcome these gaps may stimulate the upscale of these beverages in reaching wide access to contribute to the modern consumers’ quality of life.

## 1. Introduction

The traditional production of kombucha is based on the fermentation process promoted by a consortium of fungi and bacteria called SCOBY (Symbiotic Culture Of Bacteria and Yeast), using only the sugary infusion of *Camellia sinensis* leaves (black tea and/or green tea) as the mandatory substrate [1]. However, other substrates have strong promising technological potential to be used in combination with *C. sinensis*, or alone, to produce analogous fermented beverages with a wide range of sensory characteristics and functional properties.

In Brazil, the Department of Agriculture, Livestock and Food Supply (MAPA) defined kombucha as a fermented drink obtained through the aerobic and anaerobic fermentation, with SCOBY, of the must prepared through the infusion, or extract, of *Camellia sinensis* and sugars [2]. Some optional ingredients are allowed by Brazilian legislation, such as the infusion of other plant species, fruits and vegetables, spices, honey, molasses and other sugars of vegetal origin, CO_2_, fibers, vitamins, minerals, and other nutrients, in addition to natural additives, such as natural food colorants [2]. The Brazilian Normative Instruction has been established, aiming to regulate the kombucha that is subjected to technologically appropriate industrial processes and intended for human consumption as a drink.

Generally, to initiate the fermentation, sugar is added in addition to an amount of a starter inoculum composed from aliquots of any previous kombucha production [1]. The starter inoculum is responsible for lowering the pH value of the new substrate that will be subjected to the fermentation process, which will inhibit possible contaminations by undesirable microorganisms [1]. After the inoculation of the starter culture in the *Camellia sinensis* substrate, inoculation with SCOBY proceeds, and fermentation takes place in sterilized glass jars or stainless steel covered with porous fabrics permeable to oxygen, which prevent the entry of insects or other physical contaminants [1].

The fermentation step is usually carried out for 7 to 10 days, at temperatures varying from 18 to 30 °C, and after this step, the SCOBY is removed, and the fermented liquid is transferred to glass or plastic bottles, followed by an optional step of adding flavor. Generally, kombuchas with long fermentation times result in beverages with high acidity; in addition, those with a high amount of inoculum will decrease their pH to below 4.2, with this being desired to keep their ingestion safe [3,4]. Some optional ingredients can be further added to kombucha to impart distinct flavors, such as whole fruits, fruit pulp, and spices, among others; the refrigerated kombucha can then be consumed 24–96 h after the flavor addition [3,4]. From information collected in studies with consumers of this type of food product, the consumers answered that they expect that kombucha would be sold bottled in glass, under refrigeration, flavored, and not pasteurized [4].

Historically, the health benefits associated with the consumption of kombucha appeared along with the first such research in the 1970s, and its popularization in the 1990s boosted its sales. Reports from frequent consumers who have consumed kombucha since 1998 claimed improved hair, skin, and nail health; a reduction in insomnia; relief from migraines; a reduction in kidney stones; a reduction in menstrual discomforts and hot flashes during menopause; an improvement in vision; relief from bronchitis and asthma; and an increase in general metabolism [5,6,7,8].

Considering the promising technological potential related to the use of other substrates to produce fermented beverages analogous to kombucha, the Amazonia biome rises as one of the richest biodiversity locations in the world for providing plant sources as alternative substrates. The Amazon biome hosts the largest continuous extensions of tropical rainforests in the world, being a biome of great importance for the maintenance of terrestrial life, and its flora offers the possibility of various studies in different areas such as medicine, nutrition, and economics, among others [9]. It is estimated that around 60,000 species of plants are in the Amazon region, according to IBAMA [10], and a number of these plants still have unknown chemical and medicinal properties due to several factors, such as their location, a lack of incentive in scientific research, or little inclusion of these materials in the development of new products and new drugs [9,10].

However, the scientific concern for investigating the chemical composition of medicinal herbs and traditional and non-conventional vegetables has increased due to their beneficial potential and unique properties that can help combat several human diseases and due to the promising option of including their plant extracts in the development of new products, drugs, and foods, thereby adding value to the Amazon region [11].

Many people still turn to herbs or medicinal preparations as an instant therapeutic resource to treat illnesses. Studies of interest in herbs and vegetables from the Amazon region exist and have been carried out due to the incorporation of bioactive compounds of interest into foods, beverages, and herbal products [12,13]. These elements can assist in metabolism or be of paramount importance in the development of further related studies [9,14].

Studies on the levels of bioactive compounds found in Amazonian herbs have great potential to be explored due to the range of species present in the biome [15], such as the presence of bioactive phytochemicals, the total content of phenolic compounds, the total flavonoids, and the antioxidant capacity of the infusions of these plants [16]. Some studies have shown that the levels of phenolic compounds ranged from 1.7 to 83.22 mg of gallic acid equivalent/g in the following Amazonian species: Agirú (*Chrysobalanus icaco*), Açoita-cavalo (*Luehea speciosa*), Capim-santo (*Lippia alba*), Graviola (*Annona muricata*), Jucá (*Caesalpinia ferrea*), Pata-de-vaca (*Bauhinia forticata*), Parirí (*Arrabidaea chica*), Common sacaca (*Croton* spp.), *Matricaria chamomilla*, *Echinodorus grandiflorus*, *Artemisia absinthium* L., *Baccharis trimera*, and *Acmella oleracea* [9,17,18,19,20,21].

The high content of phenolic compounds found in some infusions can be a part of several mechanisms of action that promote health promotion, such as maintaining hepatic and hypolipidemic activity [22]. One of the greatest benefits still occurs in the evaluation of antioxidant activity, and herbs such as agirú and jucá, for example, achieved moderate antioxidant activity [9,23]. Therefore, not only are the flavors supposed to change of fermented beverages, such as kombucha and analogous drinks, according to the type of substrate, species of microorganism, fermentation conditions, and flavor addition but also the chemical composition and biological potential. Thus, alternative substrates would expand the visibility of kombucha and analogous fermented beverages, stimulating the research on and food technology development of fermented drinks, in addition to adding potential health benefits and increasing the product’s annual economic growth rate. 

Therefore, this review gathers information regarding promising alternative substrates to produce fermented beverages analogous to kombucha, focusing on plants available in the Amazonia biome, as alternative plant infusions are expected to increase the opportunity to develop new food products with high technological potential and functional properties.

## 2. Kombucha Popularization and Commercialization 

Since ancient times, kombucha has been mentioned, whether as a detoxifier in China in 220 BC, as a cure for digestive problems in Korea and Japan in 440 BC, or as a medicinal treatment in Russia in the 1800s AD [5,24]. However, only in recent years has the visibility of the research, development, and sales of kombucha been receiving attention from the scientific community due to the popularization of the non-commercial sharing of the consortium of microorganisms responsible for kombucha fermentation (SCOBY).

The industrialization of kombucha began in the 1990s in California (USA) and soon, after the recognition of the beverage, several companies began to establish themselves in the market until legislation regulated the production of the drink in 2014. After gaining popularity in the 1990s, kombucha production reached the level of about USD 1.8 billion in the world market and USD 1.67 billion in 2019, with an annual growth rate of 23% in market share (2014 to 2018), and this number does not include companies that are not legally registered [3,25]. The United States and Canada are the countries with the highest number of companies that produce kombucha, but other countries also have a high number of companies such as Mexico, Spain, and Australia [3].

In Brazil, kombucha legislation was established in 2019 and its popularization began with the Zanlorezi family; they started putting kombucha on shelves in the second half of 2018 with their *Campo Largo* brand. The Zanlorezi family observed a 5% drop in the soft drink market while kombucha’s share rose as a healthy alternative [26]. In Brazil, *Companhia dos Fermentados* and *K. Probioticos Artesanais* can be mentioned as pioneers in the production of kombucha, which started as an artisanal product until reaching the industrial scale [26,27]. According to the Brazilian Kombucha Association, about 40 companies contribute, with an average production of 2 to 5 thousand bottles per month, generating around BRL 20 million (≈USD 100 million) in sales in 2018 [4,28,29].

The global increase in demand for alternative and healthier food products has been increasing the number of individuals who make and consume Kombucha, as well as the creation of small companies that carry out production on semi-industrial scales. The continuation of this chain stimulates the creation of larger companies with production at industrial levels, making it easier to find kombucha in natural food chains and supermarkets [3]. Thus, frequent concerns from consumers for health contributed greatly to kombucha advancing in the food market since functional and medicinal drinks create opportunities for the industry’s economy. The future of these types of food products will be determined by their effectiveness in promoting health, new marketing strategies, and effective strategies to improve their sensory characteristics [30]. 

## 3. Strategies Adopted for Gathering Information in the Available Literature

This integrative review was elaborated based on the scientific research available in the literature containing published data related to the interested topic. The steps followed to construct this review were the recognition of the interested topic, the formulation of the research question, data collection, the categorization of studies, the analysis and interpretation, the presentation of the results, and the conclusion [31,32]. The PICO strategy [33,34] was used to guide our research: the defined population included researchers and producers of kombucha and its fermentation methods (P); the variable of interest was defined as alternative substrates for fermented beverages analogous to kombucha (I), and the context was the elaboration of new fermented products for human consumption (CO). Furthermore, the question that guided this research was as follows: “What are the new alternative substrates and their elaboration technologies for developing new fermented beverages analogous to kombucha for human consumption?”.

The literature search in the scientific databases was carried out using the term “kombucha tea” combined with the terms “substrates” and “based”, which procured 13,008 results in the following databases: Google Scholar (10,320), Scopus (1490), ScienceDirect (Elsevier) (1132), and PubMed (66). The inclusion criteria were original peer-reviewed articles, theses, dissertations, abstracts, and works published between 2003 and 2023 in English, Spanish, or Portuguese; these needed to have been made available in their full texts, addressing the use of alternative substrates to *Camellia sinensis* for the development of new fermented beverages analogous to kombucha and their production technologies. The relevant items used for data collection and analysis were the publication title, author(s), objectives, year of publication, journal, type of substrate, methodologies, and bioactive potential. At the end of the eligibility process, data collection was carried out through the selection of works by agreeing with the objective of the review.

**Figure 1:**
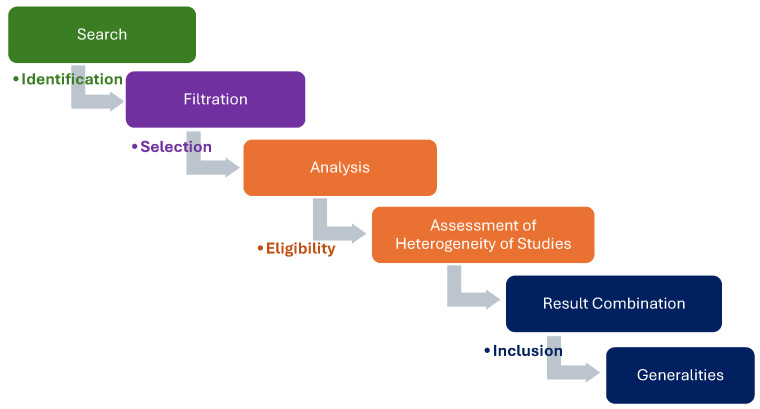
Prisma Methodological Process Flowchart.

In the pre-analysis, the selected articles were studied and the results were processed, interpreted, organized, and according to the main elements, the questioning categories were identified. In stage II (analysis), the coding, classification, and aggregation of the data found were carried out. Finally, the search strategies used in the databases to obtain the results included in this review can be seen in the Prisma Methodological Process Flowchart (Figure 1).

## 4. Results and Discussion

### 4.1. Identification of Alternative Substrates for the Production of Fermented Beverages Analogous to Kombucha

After applying our strategies for gathering information available in the literature, 13,009 primary studies were identified in the scientific databases. The search strategies used after identification to exclude papers were selection, eligibility, and inclusion, with 3748 works being excluded for not meeting the inclusion criteria, 1981 due to the repetition of information, 7247 articles being excluded in the analysis stage due to ineligibility, and, finally, 8 articles were excluded in the last stage of inclusion. These steps were carried out according to the recommendations made by the Prisma group (Main Items for Reporting Systematic Reviews and Meta-Analyses), which is characterized by a checklist of data collection items and a four-phase article selection flow diagram [35], as shown in Figure 1.

From crossing the descriptors in the databases, 36 works were selected for full reading, and, in the final stage, 29 answered the guiding question that highlighted 37 alternative substrates for the discussion of this review. Table 1 was prepared presenting the particularities described according to the alternative substrate, preparation methodology, and main results. Moreover, special focus was directed to the plants available in the Amazon region that may show sensorial acceptance and market potential to be inserted into manufacturing lines.

From the selected publications, the following information can be inferred: 67.8% of the drinks use sucrose as the carbon/energy source, with other sources being fructose, glucose, and lactose and 60.7% prepared infusions at temperatures ranging from 90 °C to 100 °C, which allowed for the extraction of hydrophilic bioactive components, mainly phenolic compounds, to compose the alternative substrates. The other studies did not specify or apply heating for the infusion preparation. Concerning the fermentation step, we observed that the controlled conditions were quite heterogeneous among the selected literature, with the highest occurrence found for the 14-day fermentation time, appearing in approximately 28% of the studies. In addition, the temperatures at 25 °C and 28 °C were the most used to carry out the fermentation processes. Furthermore, a very high percentage of the selected works (96%) did not carry out or state if a second fermentation step was performed, with the production of the fermented products being analogous to kombucha described only by one fermentation step with controlled temperature and time conditions.

### 4.2. Bioactive Compounds and Potential Advantages of Using the Selected Alternative Substrates in the Production of Fermented Beverages Analogous to Kombucha

Traditional kombucha is a good source of bioactive compounds, and the claimed health benefits associated with its consumption are associated with the presence of phenolic compounds from *Camellia sinensis*, and organic acids, mainly glucuronic acid, formed during fermentation. Several phenolic compounds were already identified in kombucha prepared with *Camellia sinensis* leaves, and the most abundant ones in the literature are catechins such as gallocatechin 3-*O*-gallate/epigallocatechin 3-*O* gallate, gallocatechin isomer/epigallocatechin, catechin, 5-*O* galloylquinic acid, and quercetin derivatives such as quercetin 3-*O*-rhamnosyl-rhamnosyl glucoside and quercetin 3-*O*-glucosyl-rhamnosyl galactoside [65]. Importantly, the number of bioactive compounds in these types of fermented beverages will vary according to the scale of production and proportion of ingredients in the formulation, such as dried herbs, sugar, and water; additionally, possible adverse effects on human health must be systematically investigated [66].

Considering all the phytochemical differences among the potential alternative plants to produce fermented products analogous to kombucha, not every substrate will present the same composition, biological potential, or sensory characteristics after being subjected to fermentation. For instance, depending on the bioactive compound composition, one substrate may exhibit higher microbial activity than antioxidant capacity and vice versa, while another one may show hypolipidemic or glycemic properties. Thus, all the potential health benefits observed for each new substrate do not exclude the results obtained from the others, reiterating the need to develop systematic studies on alternative substrates, including the sensory acceptance on the part of kombucha (and analogous beverage) consumers.

During the fermentation process, the acidic conditions in aqueous medium induce the formation of simpler phenolic compounds because cleavages of more complex structures. Some substrates such as acerola cherry, Mate herb, and chamomile pointed to the biotransformation that generally occurs with *C. sinensis*, which was epigallocatechin gallate being transformed into isomers such as epicatechin and epicatechin gallate, which are smaller molecules [67,68].

The amount of ascorbic acid also increased during the fermentation of the acerola cherry kombucha-type beverage [40]. Monitoring the kinetics of kombucha fermentation is a complex process due to the variety of active microorganisms and their interactions, resulting in the production of several compounds, among which the following stand out: gluconic, glucuronic, lactic, acetic, malic, tartaric, citric, and oxalic acids [65]. In addition, there is the production of ethanol, various amino acids, water-soluble vitamins, and hydrolytic enzymes.

In some studies with traditional kombuchas, 127 phenolic compounds were identified, of which 70.2% were flavonoids, 18.3% were phenolic acids, 8.4% were polyphenols, 2.3% were lignans, and 0.8% were stilbenes [1,65,67]. In 2020, analyses of traditional kombuchas brought to the literature 103 phenolic compounds that had never been previously reported before [65].

The use of alternative substrates to produce fermented beverages analogous to kombucha, for instance, chamomile, hibiscus, lemongrass, American tarwort, and malvaviscus, positively contributed to adding high contents of bioactive compounds, high antioxidant, and antimicrobial properties [57,69].

As can be seen in Table 1, 37 alternative substrates were shown. In Brazil, *Hibiscus sabdariffa* L. flowers are widely available in the Amazon region, with its widespread use within the states of Maranhão, Amazonas, Acre, Roraima, and Rondônia, and its use in cooking is quite popular. *Hibiscus sabdariffa* L. flowers have stood out for their antioxidant compounds such as vitamin C, anthocyanins, flavonoids, phenolic acids, and β-carotene, among others, which have been attributed to many of their health benefits [37].

Acerola cherry (*Malpiguia emarginata*) is a tropical fruit with a high potential to be used as a substrate that is also widespread in the Amazonia biome, being mainly used to produce artisanal juices, sweets, and jellies. Acerola cherry is considered a “superfruit” due to its vitamin C and polyphenol contents. These compounds have been associated with some biological activities related to the reduction in oxidative stress. Just like acerola cherry, byproducts from the fruit processing industry also contain a large number of bioactive compounds, which are quantified via analyses of the content of phenolic compounds, antioxidant activity, and vitamin C.

Lemon balm (*Melissa officinalis*) is a plant native to South-Central Europe, the Mediterranean Basin, Iran, and Central Asia, but now naturalized elsewhere, whose infusion is widely used in the Amazon region for various purposes, for example, by women in labor. The main phenolic compounds quantified for lemon balm were gallic acid, catequins, epicathequins, rutins, and coumaric acid [44].

Malvaviscus (*Malvaviscus arboreus*) and mint (*Mentha piperita*) also have their infusions used within Amazonian communities but they are less popular than lemon balm. Malvaviscus flowers have a percentage of 43% of their compounds being anthocyanins, which are responsible for the characteristic color and for the antioxidant activity of this kombucha; other bioactive compounds include protocathequin acid, ferulic acid, synapinic acid, myricetin, and quercetin [57].

Rosemary (*Rosmarinus officinalis*) and fennel (*Foeniculum vulgare*) are herbs used in the Amazonian community for fevers, dyspepsia, cardiac weakness, hipsterism, and nervousness. Nettle (*Urtica dioica*) is used to maintain the circulatory system, being antibacterial and a diuretic depurative. Peppermint is also used in the region as an antispasmodic and is used extensively in products due to its menthol and tripertene derivatives [45,56].

Papaya fruit (*Carica papaya*) has been the target of research in the Amazon region, initiated by the Brazilian Agricultural Research Corporation (EMBRAPA—*Empresa Brasileira de Pesquisa Agropecuária* in Portuguese), which aims to adapt the technological, production, nutritional, and sensorial quality of Brazilian fruits to the international scene, thereby increasing the competitiveness of Brazil abroad [59]. *Chenopodium ambrosioides L.* is also planted and harvested in the Amazon region and is popularly known for its anthelmintic action and for flu treatments; studies have reported on the biological activities of this species, such as antitumor, antipyretic, analgesic, antifungal, anthelmintic, and leishmanicidal activities [64]. 

Therefore, among all the alternative substrates listed in Table 1, approximately 27% are easily found in the Amazonia and they were used successfully to produce fermented beverages analogous to kombucha even though they are not native to this biome.

### 4.3. Functional Properties and Biological Activity of Fermented Beverages Analogous to Kombucha

Regarding the beneficial biological properties of kombucha, kombucha was reported to reduce glycosylated hemoglobin, increase plasma insulin and tissue glycogen levels in diabetic rats, and regulate the enzymatic activities inherent in glucose regulation routes [70]. The frequent consumption of kombucha was reported to decrease the risk of the development of cardiovascular diseases, inhibit LDL oxidation, regulate cholesterol metabolism, and prevent high blood pressure [5,71]. D-Glucuronic acid, which can be found in kombucha, plays a role in xenobiotic liver detoxification and endobiotic elimination, thereby enhancing liver functions [72]. Another study showed that kombucha prepared with lemon balm was not genotoxic and presented antigenotoxic effects, in addition to affecting the cell growth of HeLa, MCF7, and HT-29 human tumor cell lines, but this kombucha did not achieve 50%-cell growth inhibition [73]. The claimed benefits of consuming kombucha include improving liver function [74], the immune system, gastrointestinal functions [53,75], and anti-inflammatory properties [76], in addition to reducing cholesterol levels and blood pressure [77].

One of the main biological properties associated with kombucha is its antimicrobial activity, which is attributed to its acidic pH, due to the presence of organic acids, such as acetic acid, and phenolic compounds, such as catechins, that inhibit the growth of a series of Gram-positive and Gram-negative bacteria [53,73]. Kombucha is claimed to strengthen the immune system [75], acting similarly to probiotic products [24], but this association cannot be used for commercial purposes, as this property was not yet scientifically proven in human trials. For the same commercial reasons, Brazilian legislation does not allow labels that use expressions of superlative qualities characteristics, and/or functional properties, which are not approved by specific legislation, such as artisanal, homemade, family, live drink, probiotic drink, ancient drink, elixir, elixir of life, energizing, invigorating, special, premium, among others [78].

Regarding studies related to the potential health benefits of fermented beverages analogous to kombucha in the selected articles, we observed that 21 papers reported the antioxidant capacity determination, and most of them (78%) were carried out by scavenging the DPPH radical, which is a synthetic and stable free radical not existing in the physiological system. Other antioxidant capacity assays commonly used by the scientific community seem to be scarce, such as the scavenging capacity against ABTS radical, which is another synthetic and stable free radical not found in the physiological system.

Mendonça et al. [79] pointed out 15 articles reporting antioxidant capacity results as the main approach among 18 articles, and 7 presented antimicrobial assays. However, to the best of our knowledge, the evaluation of the antioxidant potential of kombucha or kombucha-type drinks concerning the scavenging capacity against reactive oxygen species (ROS) and reactive nitrogen species (RNS) of physiological and food relevance was not yet reported in the literature. This fact suggests the need for more in-depth systematic studies to increase the understanding of the antioxidant potential of this type of fermented food.

To confirm not only the effects of traditional kombucha but also those of analogues that contain alternative substrates in their composition, existing in vitro and in vivo studies have been growing increasingly [80]. By 2020, approximately 98 clinical evidence studies were quantified, in which biological activities were analyzed in experimental tests to check the allegation efficiency.

Antioxidant assays were presented in approximately six in vivo and three in vitro assays; immuno-modulatory activities were presented in approximately four in vitro assays; antitumor drugs were presented in eight clinical evidence studies; hypolipidemic activities were presented in four in vivo trials; hypertension control activities were presented in a clinical trial with humans; activities related to glycemia were reported in approximately three in vivo and six in vitro studies; antimicrobial trials were in two in vivo studies; and neuroprotective activities were reported in two in vivo assays and two in vitro assays [80].

In general, the percentage of studies with new alternative substrates is lower than studies carried out with traditional infusions. Therefore, in vitro and in vivo investigations are of paramount importance to strengthen the association between bioactive compounds and the claimed biological effects associated with health, in addition to the bioaccessibility and bioavailability determinations for these compounds.

Eight articles selected for this integrative review evaluated the production of organic acids with the SCOBY; the main acids reported were ascorbic, acetic, and glucuronic acids and the main conclusion was that they display biological properties, such as hepaprotective effects, but the hardest part is to associate clear biological effects to one of the organic acids, such as glucuronic acid in case of hepaprotective effects, due the lack of studies that can prove this without a margin of error [72].

Several studies with various substrates in addition to the traditional ones, such as berries and lemon balm, which listed organic acids such as acetic, glucuronic, oxalic, formic, succinic, citric, and malic acids, were also present in the Amazon region and have been explored in these last few years. The identification of the microorganisms present in the SCOBY and the metabolic biotransformation pathways already identified in Kombucha also need continuous studies to understand the production of these organic acids. This type of approach would help us understand and predict the composition and biological potential of bioactive compounds contained in the ready-made drink at the time of consumption.

Nine articles evaluated antimicrobial activity, most of them via the inhibition halo assay. Antimicrobial inhibition was attributed to the production of organic acids during fermentation as well as the phenolic compounds found in the substrate, such as catechins. As an example, one in vitro study in 2018 used yarrow (*Achilea millefolium*) as a substrate and observed an inhibitory effect not only on pathogenic microorganisms but also on cancer cells such as rhabdomyosarcoma, which is derived from cervical carcinoma, and rodent fibroblast cells [63].

In the literature, the kombucha made with *C. sinensis* showed positive results against *S. aureus*, while black tea was able to inhibit the growth of *E. coli*, and the ones made with alternative substrates, such as lemon balm, red grape juice, and garlic also showed inhibition against Gram-positive bacteria and *S. aureus* [79]

One article monitored the biofilm formed with the SCOBY using the SEM (Scanning Electron Microscopy) method and concluded that the substrate prepared with residues of acerola cherries stimulated the formation of new SCOBYs that exceeded expectations, thus requiring further studies that led to new substrate characterization [41].

Other examples of reported biological properties were also observed for kombucha analogues such as the inhibition of acetylcholinesterase and xanthine oxidase, while two articles showed cytotoxic activity. Another study evaluated the performance of anti-α-glucosity and anti-α-amylase agents, and two articles evaluated the inhibitory activity of starch hydrolase.

Three articles selected to construct this review presented only physical–chemical characterizations such as pH values, titratable acid content, Brix value, sugars, and ethanolic content, which are extremely important, as it is through physical–chemical evaluations that the safety of ingesting the drink can be attested and correlate the effects to the bioactive compounds found in each substrate used.

Fermented drinks are known to regulate the gut microbiota and establish inhibitory competition with pathogens, both due to the acids produced during the fermentative processes and the microbial activity of the drink. Among the nine articles that evaluated this activity, the main microorganisms inhibited were *B. cereus*, *S. typhimurium*, *E. coli*, and *S. aureus.*

The microbiological inhibitions studied in kombucha have their limitations; even though the fermented product influences many pathogens, studies in the area present a variability of results in relation to the microorganism exposed to the inhibitory action, that is, the studies will not always test the inhibition in the same microorganism.

However, the type of substrate and concentrations of them or their extracts for observing this microbial activity also differ. These limitations are not necessarily negative, as they corroborate the inclusion of more substrates that demonstrate the inhibition of pathogens that have not yet been inhibited by existing substrates.

In light of the above, it appears that studies with kombucha and the development of alternative substrates present many variables to be considered when investigating the functional aspect of the drink. This variability concerns the microorganisms present in the SCOBY, the fermentation conditions, and the phytochemical composition of the different substrates. Furthermore, the content of bioactive compounds may change during the process, modulating the potential biological properties.

### 4.4. Toxicity of the Fermented Beverages Analogous to Kombucha

Concerning the adverse effects, Jayabalan et al. [24] reviewed cases of toxicity in the literature associated with kombucha and reported nausea, dizziness, and other severe symptoms, leading to poisoning (from specific recipients), gastrointestinal toxicity, contamination with *Bacillus anthracis* due to poor hygienic conditions, and worsening health conditions in HIV-positive people. These same authors also reported side effects such as allergies, vomiting, headache, and neck pain due to kombucha consumption. These and another few cases led the FDA (Food and Drug Administration) to carry out safety tests on kombucha, and the agency certified that it is not toxic, and the side effects related to excessive consumption are isolated events, only being contraindicated for pregnant and breastfeeding women [24]. As every new food product must be systematically investigated regarding its toxicity to humans, the toxicity of the fermented beverages analogous to kombucha must also be carefully observed, especially in the case of the use of underexploited or scientifically unknown plant sources.

### 4.5. Sensory Aspects of Fermented Beverages Analogous to Kombucha and Market Prospection

Regarding the fermented beverages analogous to kombucha produced with alternative substrates to the market, and the inclusion of these new substrates in the production line, only about 23% of the selected articles addressed sensory acceptance tests. Importantly, the development of new food products with functional claims also needs to present desirable sensory characteristics to encourage frequent consumption and the adoption of healthier habits. Given that the global and Brazilian markets are still popularizing kombucha, there is a need for additional studies comprising the economic sector in which this drink fits and bringing about the inclusion of these alternative substrates, increasing the variety in the market and availability.

The alternative substrates native to or adapted into the Amazonia biome are already present in the data collection with results that require continuity. As already stated, the development of these new food products stimulates the valorization of the production chain of underutilized raw materials, moving the national bioeconomy.

## 5. Conclusions

This review highlighted that there is a growing increase in the development of fermented beverages analogous to kombucha with alternative substrates as replacements for *C. sinensis*, by focusing on new or improved technological and functional properties. Alternative substrates of plants from the Amazonia biome, or any other biome, rise as promising sources of bioactive compounds, such as phenolic compounds, which are expected to add relevant biological properties to the final product depending on the type and concentration of each plant source. Furthermore, both the control and standardization of the beverage production process and the sensory acceptance approaches need to work together; otherwise, it becomes unfeasible for alternative substrates to be commercially competitive in comparison to traditional substrates.

Among the many aspects that still require systematic investigation to better understand the functional potential of fermented beverages analogous to kombucha, we can mention several gaps to be filled, such as detailed studies about the bioactive compound compositions of promising plant sources and derived formulations; studies about antioxidant capacity (in vitro and in vivo) by scavenging reactive pro-oxidant species of physiological relevance; the determination of in vivo enzymatic modulation; the monitoring of the transformation of bioactive compounds in the presence of SCOBY in the intestinal microbiota; and clinical interventional studies concerning the effect of the frequent intake of kombucha-type beverages on the development of chronic noncommunicable diseases.

## Figures and Tables

**Table 1:** Alternative substrates as replacements of *Camellia sinensis* infusions to produce fermented beverages analogous to kombucha, fermentation conditions, and main results.

Raw Material	Infusion/Preparation	Fermentation	Main Results	References
Hibiscus (*Hibiscus sabdariffa* L.)	Dehydrated flowers (1%, *w*/*v*) and commercial sucrose (8%, *w*/*v*), in water at 100 °C. SCOBY (10%, *w*/*v*) was added to the filtered and cooled infusion (25 °C).	1st fermentation: 14 days at 25 °C. 2nd fermentation: After 1st fermentation, SCOBY was removed and the fermented infusion was kept for 12 days at 25 °C.	Sensory-accepted drink with characteristics of the raw material present, acidity lower than expected, and presence of anthocyanins in the final product. Kombucha-type product with higher antioxidant potential than traditional ones.	[36,37]
Red and Black Goji Berry *Lycium barbarum* and *L. ruthenicum*	Infusion prepared with 1% Black Goji berry or 1% Red Goji berry in water at 98 °C/15 min, before the addition of 6% sucrose.	2 days at 28 °C.	All kombucha types prepared with goji berry showed higher antioxidant capacity than traditional infusions, with black goji berry being the substrate with the highest phenolic compound contents and antioxidant capacity.	[38]
Grape Juice (*Vitis vinífera* L.)	Grape juice (25%) + 3% of SCOBY and + 10% of starter.	12 days at 30 °C	Fermented drink with high antioxidant potential associated with the presence of phenolic compounds generated by the microbial action on grape juice. Antimicrobial activity against Gram-positive and Gram-negative pathogenic bacteria was observed.	[39]
Acerola Cherry (*Malpiguia emarginata*) byproduct (pulp from clarification process.)	1, 3, and 5% acerola byproduct (dry basis) + 35 g/L glucose and 35 g/L fructose + 10% starter. 12, 36, and 60 g of dried acerola byproduct + 600 mL of distilled water; extraction at 50 °C, 150 rpm/4 h. 207 mL of extract + 198 mL of Sucrose + 18 g of SCOBY and + 45 mL of starter.	15 days at 30 °C. 21 days at 30 °C.	Increased phenolic and vitamin C content in the product. The content of bioactive compounds accelerated the metabolism of microorganisms, increasing substrate consumption and the production of ethanol, acetic acid, and bacterial cellulose.	[40,41]
Milk	Fermented drinks produced with milk with 2.0% fat and 10% inoculum, at 37 °C and 42 °C, up to a pH of 4.6.	The study had kombuchas prepared under 3 fermentation times: 1—37 °C—13 h and 40 min. 2—42 °C—8 h and 15 min. 3—42 °C for 14 days.	It showed high antioxidant activity during storage. Good sensory acceptance for appearance, flavor, and overall impression.	[42]
Rooibos (*Aspalathus linearis*)	8 g/L at 95 °C for 10 min + 80 g/L of sucrose and 30 g of SCOBY.	27 °C for 14 days	Increased antioxidant activity in relation to its initial capacity before fermentation and traditional infusions. Decrease in the content of total phenolic compounds in the product.	[43]
Lemonbalm (*Melissa officinalis*)	70 g of sucrose in 1 L of boiling water + 5 g/L of Lemon balm + 100 g/L of SCOBY + 0.33 L of inoculum.	28 °C for 7 days	Production of a drink with antioxidant properties (action of rosmaric acid) and antimicrobial properties (Gram-positive and -negative bacteria) superior to traditional infusions.	[44]
Mint (*Mentha piperita*) Thyme (*Thymus vulgaris*) Lúcia Lima (*Lippia citriodora*) Rosemary (*Rosmarinus officinalis*) Fennel (*Foeniculum vulgare*)	10 g of dry herb + 20 g of sucrose in 1 L of boiled water, infused for 15 min. After filtration, 250 mL of the filtrate is added with 10 g/L of SCOBY.	21 days at room temperature (25 °C)	High performance of catechins in microbial inhibition in Gram-positive and Gram-negative bacteria and in strains of *Candida*, except *C. Crusei* for all analogues prepared.	[45]
African Mustard (*Brassica tournefortii*)	77 g of sucrose + 1100 mL of water and heated at 90 °C + 10.5 g of leaves for 15 min. In 350 mL, 7 g of SCOOBY was added.	25 °C for 14 days	Increased antioxidant activity and total phenolic compounds. Decrease in xanthine oxidase activity and cytotoxic action of leaves.	[46]
Snake Fruit (*Salacca zalacca Gaerth. Voss*)	400 g of snake fruit was crushed in water in a ratio of 1:1 (*w*/*w*) and after filtering they was sweetened in a ratio of 1:10 (*w*/*v*); after pasteurization, the juice was inoculated with starter in a ratio of 1:10 (*w*/*w*).	14 days at room temperature	Increased antioxidant activity, total phenolics, total flavonoids, and total tannins in relation to their initial content and traditional kombuchas.	[47]
Pear/Prickly Pear Cactus Juice (*Opuntia fícus indica*)	300 mL of Pear Cactus juice + 3% SCOBY + 10% starter.	12 days at 30 °C	Increased antioxidant activity and CFT. It showed antimicrobial activity against Gram-negative pathogenic strains such as *E.coli* and *Bacillus cereus*.	[48]
Whey/Cheese Whey	70 g/L sucrose + 20 g SCOBY + 250 mL cheese whey/milk.	32 °C for 4 days	Identification of acetic bacteria (*Gluconoacetobacter xylinus*) and fungi (*Saccharomyces cerevisiae* and *Brettanomyces* *Bruxelensis*), changes in the structure/surface of the colony due to the substrate.	[49]
Zijuan (*Camellia sinensis var. kitamura*)	1.5 of water + 120 g of sucrose + 9 g of herb infused at 90 °C for 10 min. Inoculated with 5% starter.	28 °C for 14 days	Zijuan Kombucha presented greater sensorial acceptance than traditional ones, as well as having greater antioxidant power due to the presence of catechins and higher concentrations of volatile compounds.	[50]
Soy Drink (*Glycine max.*)	5% starter with 10 days of cultivation in 1000 mL of soy drink.	28 °C for 4 days 37 °C for 3 days	Formation of isoflavone aglycone by hydrolysis, increase in phenolic compounds and vitamin C, increase in antioxidant potential.	[51]
Soy Whey (*Glycine max.*)	1:10 (inoculum/soy drink, *v*/*v*) For every 100 mL used in the inoculum, 0.39 of crude fat, 0.71 g of protein, and 0.39 of ash were obtained.	28 °C for 8 days	6 days of fermentation are the best conditions for elaboration, with excellent antioxidant potential and antimicrobial activity due to the content of aglycone isoflavones and other volatile compounds.	[52]
Coffee (*Coffea arabica*)	1 g of coffee added to every 100 mL of water + 10% sucrose + 3% starter + SCOBY.	7 days at 24 °C	High antioxidant potential, increase in α-amylase inhibitory activity during the fermentative process.	[53]
Garlic and Vinegar (*Allium sativum*)	150 g +300 mL of red grape vinegar and left for 1 month. 10 g of extract + 20 g of sucrose in 1 L, boiled for 5 min, and cooled for 15 min, after which the SCOBY was added.	21 days at 37 °C	Gallic acid increases as the fermentation process progresses, resulting in an increase in antioxidant potential. Decreased antimicrobial activity (Gram-positive bacteria) of garlic extracts is achieved after fermentation.	[54]
Mate Herb (*Illex paraguasiensis*)	Erlenmeyer flask (500 mL) was added to 270 mL of the culture medium prepared at the yerba mate concentration established for each test + 5% sucrose + 30 mL of starter.	20 °C for 12 days	Drinks with 1% yerba mate at 20 °C and 1% yerba mate at 30 °C showed a higher concentration of phenolic compounds and, consequently, greater antioxidant activity. They showed antibacterial activity, being effective in inhibiting the growth of strains of Staphylococcus aureus and *Escherichia coli*, as well as inhibiting oxidative stress in vivo using the microorganism Saccharomyces cerevisiae, being able to increase the survival rate of yeast against H_2_O_2_ as stressor agent.	[55]
Savory (*Satureja montana*) Nettle (*Urtica dioica*) Serpilho (*Thymus serpyllum*) Elderberry (*Sambucus nigra*) Peppermint (*Mentha piperita*) Marmelo (*Cydonia oblonga*)	1 L of boiling water + herbs (3 g for elderberries, the others were prepared with 2.26 g) + 70 g of sucrose; in a 600 mL bottle the substrate +10% SCOBY was added	0, 3, 7, and 10 days of fermentation at 25 °C	These herbs were able to produce a greater metabolic activity than the traditional substrate when analyzing the production of organic compounds; the herbs showed superior oxidizing power; peppermint and traditional. Green Tea and Serpilho were the largest that eliminated the DPPH radical. Savory was the greatest that eliminated the hydroxyl radical. In activities against hypertension, all of them have excellent performance, especially elderberry and nettle.	[56]
Malvaviscus (*Malvaviscus arboreus*)	Infusions (0.5%, *w*/*v*), + sucrose (5.0%, *w*/*v*) + SCOBY (2.5%, *w*/*v*) + 1.0% (*v*/*v*) starter	24 ± 2 °C for 14 days	A well-fermented product was produced that showed excellent antioxidative potential.	[57]
Corn Silk	5 g of corn silk + 100 mL of boiling water at 100 °C for 15 min. 500 mL of the filtrate + 100 g granulated white sugar + 30% of starter.	28 °C for 7 days	Production of a drink within the standards for a fermented Kombucha type with a tendency to have good sensory performance.	[58]
Papaya (*Carica papaya*)	10% dry papaya leaves/fruit + 10% sugar + 20% starter, leaving 60% for water.	37 °C for 4 days	2 microorganisms were identified in the study and these microorganisms are pathogen inhibitors.	[59]
*Porphrya dentata* (Waterplant)	50 g of sugar diluted in 1000 mL of water + water extract (60 mL) + 24 g of SCOBY.	25 °C for 14 days	Several bioactive compounds were identified, such as total phenolic compounds and flavonoids, even though it presents lower antioxidant activity than traditional kombuchas; there is a facilitated production of organic compounds.	[60]
Mexican Oak (*Quercus resinosa*)	10 g of herbs infused + 1000 mL of water for 10 min + 7 and 10% sucrose + 2.5% and 5% starter.	7, 9, 10, and 11 days at 25 °C and 35 °C	Qualification of several bioactive compounds (organic acids, alcohols, fatty acids, and phenolic compounds); the phenolic content is diverse, such as catechins, epicatechins, and benzoic acid.	[61]
Chrysanthemums, Honeysuckle (*Chrysanthemum morifolium* and *Lonicera japonica*), and fresh mint (*Mentha spicata*)	1000 mL of boiling water for 10 g of herbs and 50 g of sucrose + 100 mL of inoculum + SCOBY.	25 °C for 6 and 12 days of fermentation	Several bioactive compounds were identified in the work that give unique characteristics to each herb, such as alcohols, esters, acids, aldehydes, ketones, and others. The sensory work showed a greater preference for work herbs than traditional infusions, which may be related to isovaleric acid. Mint-based kombucha received greater approval, being described as the sweetest and most refreshing.	[62]
Yarrow/Carpenters’ Weed (*Achilea millefolium*)	1.13 and 2.26 g of herbs + 500 mL of boiling water for 15 min and 35 g of sucrose + 10% of the inoculum + SCOBY	25 °C for 7 days	Kombucha produced by fermentation in subcritical aqueous extracts showed greater antioxidant activity, but lower antimicrobial and antiproliferative activity compared to products obtained from infusions. Kombucha drinks produced from subcritical aqueous extracts of yarrow had the highest sensory score.	[63]
Mastruz (*Chenopodium ambroisoides* L.) with added *cupuaçu* pulp (*Theobroma gradiflorum*)	1.6 g of dry herb + 500 mL of water + 50 g of sucrose + 50 mL of starter after cooling + 5% (*w*/*v*) of SCOBY *Cupuaçu* pulp was to the second fermentation.	28 °C for 7 days in first fermentation process + 2 days in the second fermentation process	The product was accepted in sensorial tests, and in drying methods, a higher growth of SCOBY mass was observed as well as less contamination in the kiln route than in the freeze dryer.	[64]

## Data Availability

The original contributions presented in the study are included in the article, further inquiries can be directed to the corresponding author.
